# Editorial: Health systems recovery in the context of COVID-19 and protracted conflict

**DOI:** 10.3389/fpubh.2023.1205286

**Published:** 2023-05-24

**Authors:** Sohel Saikat, Duncan Selbie, Geraldine McDarby, Saqif Mustafa, Mila Petrova, Redda Seifeldin, Yu Zhang, Zsuzsanna Jakab

**Affiliations:** ^1^World Health Organization, Geneva, Switzerland; ^2^International Association of National Public Health Institutes, Paris, France

**Keywords:** health system, health policy, planning, resilience, recovery, COVID-19, essential public health functions, primary health care

The COVID-19 pandemic has revealed a lack of resilience in national health systems and flaws in the global coordination required for tackling a rapidly escalating emergency. This is against a background of chronic underinvestment in public health, encompassing emergency preparedness, and a fragmented approach to health system planning, financing and services. While some countries managed to limit the direct impacts of COVID-19, as reflected in case numbers and mortality rates, this came with significant costs including restrictions in movement, interruptions in trade, social unrest and unprecedented spending in health (1). The diversion of health system resources to COVID-19 response led to protracted disruptions of essential health services (2). The true extent of the impact on population health in many countries cannot be reliably ascertained due to basic gaps in health information systems and reporting. As we enter what has been coined a “new age of pandemics,” we must accept that even wealthy countries cannot afford to repeat this experience again (3).

The consequences of the COVID-19 pandemic have been amplified by pre-existing weaknesses in public health and health system capacities, with the greatest impact felt by the most vulnerable within our societies. The fact that the vulnerable and marginalized among us have been disproportionately affected, both physically and economically, is unacceptable (4–6). At the same time it is important to acknowledge that the vulnerable and marginalized were shouldering a greater share of the burden of disease prior to the pandemic, with higher rates of ill health coupled with greater difficulties in accessing health care (7, 8). Pre-existing austerity, high out of pocket payments for health services and reduced spending on public health have diminished the ability of our health systems to reach people most in need with the services necessary to prevent as well as treat disease. Vertical investment and programming within the health sector and across sectors have been proven to be ineffective in maintaining health services and responding to disruptive public health events. And, despite much rhetoric around social participation, the public's voice is often excluded from decisions around policy, planning and equitable investment in health services.

COVID-19 has once again laid bare what previous public health emergencies have demonstrated with painful clarity, that health is at the heart of social and economic prosperity. It has also reinforced that emergency preparedness and response needs to be integrated and delivered in synergy with other essential public health functions (9–11). As the world looks to recover from COVID-19, we must reimagine our health systems to ensure that the limited resources available can not only provide health systems capable of responding to the challenges presented by climate change, war and conflicts, emerging infectious threats, and rising rates of antimicrobial resistance and non-communicable disease, but also create systems for health that keep the most vulnerable among us well.

This Research Topic aims to consolidate global perspectives and experiences from the COVID-19 pandemic and protracted conflicts and to inform a different approach to policy, planning and practice to “build back better.” The majority of authors' teams are *not* affiliated with academic institutions. Instead, they represent actors whose primary responsibility has been to make the high-risk, high-stakes, real-world decisions that have impacted all our lives. They are offering their experiences to inform and strengthen recovery for all. Representing the learning captured from more than 60 countries during the acute phase of a global pandemic, this Research Topic demonstrates that difficult circumstances can and do create opportunities for change, both organizationally and at the service delivery level. As is clearly articulated by the varied approaches within the country case studies shared in *The use of innovative approaches to strengthen health system resilience during the COVID-19 pandemic: case studies from selected Commonwealth countries* (Mghamba et al.), there is no “one size fits all solution” to the development of health system resilience. However, the Research Topic also demonstrates that there are a number of common areas for action in support of this aim.

The importance of the Primary Health Care (PHC) approach,^1^ encompassing comprehensive integrated health services that embrace primary care and essential public health functions (EPHFs),^2^ multisectoral action for health and community engagement, in supporting health systems resilience and responding to emergencies, is a strong and common thread across many articles in the Research Topic. At the global level*, Developing technical support and strategic dialogue at the country level to achieve Primary Health Care-based health systems beyond the COVID-19 era* (Cheong Chi Mo et al.) presents the existing platform of the UHC-Partnership, which supports technical capacities in countries to strengthen primary health care. *From fragility to resilience: A systems approach to strengthen primary health care* (Lugten et al.), an article by USAID experts, presents an approach to strengthening PHC in countries using a systems approach. At the country level, *Learning from pandemic responses: informing a resilient and equitable health system recovery in Thailand* (Tangcharoensathien et al.) and *An overview of Iran's actions in response to the COVID-19 pandemic and in building health system resilience* (Gouya et al.) highlight the contribution of pre-existing investments in primary health care to resilience by creating the opportunity to leverage primary health care structures and platforms to promote more effective and, importantly, more equitable response efforts in Thailand and the I.R. of Iran respectively. These articles, as well another from Iran, *Risk communication and community engagement as an emerging pillar of health emergency management in Iran: achievements and the way forward* (Senga et al.), also highlight the importance of community engagement in supporting emergency response and building resilience.

The use of multisectoral collaboration to reach the most vulnerable is demonstrated in “*Beyond just the four walls of the clinic”: The roles of health systems caring for refugee, immigrant and migrant communities in the United States* (Abudiab et al.), while leveraging allied sectors to support COVID-19 response is a feature of *Lessons from inter-disciplinary collaboration to mitigate SARS-CoV-2 transmission in schools, Ireland, 2020/2021, to inform health systems and multisectoral recovery* (Naughton et al.).

The role of the essential public health functions is articulated in a number of articles. *Toward applying the essential public health functions for building health systems resilience: A renewed list and key enablers for operationalization* (Zhang et al.) discusses the development of a renewed list of essential public health functions for 21st century public health challenges, while exploring a number of key enablers to support operationalization. *A synthesis of concepts of resilience to inform operationalization of health systems resilience in recovery from disruptive public health events including COVID-19* (McDarby et al.) identifies the essential public health functions alongside learning systems and integrated health systems strengthening as a key action area to build resilience in recovery efforts. The application of the essential public health functions to strengthen public health capacities at the national level is explored in *A novel approach to utilizing the essential public health functions in Ireland's health system recovery and reform* (McNicholas et al.).

The development and use of health information to drive innovation and change from policy to service delivery levels was also a prominent theme. *Perspective: Lessons from COVID-19 of countries in the European region in light of findings from the health system response monitor* (Tille et al.) outlines how developing a repository to support policy knowledge transfer at the European level early in the pandemic helped to inform government policies to support and finance public health interventions. *Assessing capacities and resilience of health services during the COVID-19 pandemic: Lessons learned from use of rapid key informant surveys* (Rivas-Morello et al.) presents a low cost yet effective way to augment national health information systems to inform response and recovery that can be integrated into operational planning. *The provision and utilization of essential health services in Afghanistan during COVID-19 pandemic* (Neyazi et al.) discusses the use of health information to inform changes in service delivery, reorganizing care in response to the pandemic in Afghanistan.

Despite the title of the Research Topic, fragile, conflict and violence affected settings are under-represented. While this likely represents the difficulties in obtaining data from these regions, as articulated in *Early effects of COVID-19 on maternal and child health service disruption in Mozambique* (Augusto et al.), the lack of clear attention to these contexts with the greatest need must be addressed.

Similar to past public health emergencies and humanitarian responses, the recovery from COVID-19 presents us with a narrow opportunity to do things differently. The widespread impacts of COVID-19 have created an understandable drive to bolster emergency response capacities to ensure this never happens again. However, this drive could be better harnessed to ensure a renewed recognition of the broader responsibility of health systems—to provide quality and equitable services in routine times. For example, the role of primary health care for essential public health functions and the provision of public health services is critical to ensure timely detection, reporting and response.

This Research Topic calls us to innovate and learn in order to deliver essential health services in the most difficult of contexts. For any of this to translate into a resilient recovery, we must move beyond the empty rhetoric of “lessons learned” from past experience with emergencies. This requires an active approach to recovery that allows us to sustain and develop what has served us, rather than the passive free fall back to pre-existing levels of system and service delivery—a baseline that, if we are honest, was not really serving us.

- While investment will be required, it is as much about making smarter and better choices including:
- Drawing on and aligning all available resources to support integrated health system strengthening;
- Investing in cost effective and sustainable approaches like primary health care and the essential public health functions;
- Breaking down the siloes within and beyond the health system and the barriers between people and systems to ensure a whole-of-society approach to health in routine and emergency contexts including fragile, conflict affected settings;
- Developing public health leadership and institutions including at national and subnational levels based on lessons and best practices identified.

This active, evidence-informed approach to recovery is the only way for us to escape the chronic panic and neglect cycle of past public health emergencies and build a resilient and sustainable future for all. The window of opportunity for change is, once again, closing. How we act today will determine the costs that will be paid by us all, when the next, inevitable pandemic strikes.

## Author contributions

SS provided overall stewardship of conception as well as the acquisition of significant data informing the work as well as contributing to manuscript revision. DS and ZJ provided approval for conceptualization and design of the work including approval for publication of content. GM provided substantial contributions to the drafting and revising of the manuscript from conception to delivery. RS provided substantial contributions to the intellectual content of the manuscript and contributed to the revising of the manuscript and was responsible for the acquisition of significant data informing the work. YZ, SM, and MP provided substantial contributions to the intellectual content of the manuscript and contributed to the revising of the manuscript. All authors contributed to the article and approved the submitted version.

## Footnotes

^1^Primary health care is a whole-of-society approach to effectively organize and strengthen national health systems to bring services for health and wellbeing closer to communities.

^2^The essential public health functions are a set of fundamental, interconnected and interdependent activities, both within and beyond the health sector, required to ensure effective public health actions and services.
